# Vitreous Olink proteomics reveals inflammatory biomarkers for diagnosis and prognosis of traumatic proliferative vitreoretinopathy

**DOI:** 10.3389/fimmu.2024.1355314

**Published:** 2024-02-22

**Authors:** Haixia Guo, Tian Wang, Jinguo Yu, Zhemin Shi, Minghui Liang, Siyue Chen, Tiangeng He, Hua Yan

**Affiliations:** ^1^ Department of Ophthalmology, Tianjin Medical University General Hospital, Tianjin, China; ^2^ Shaanxi Eye Hospital, Xi’an People’s Hospital (Xi’an Fourth Hospital), Affiliated People’s Hospital of Northwest University, Xi’an, Shaanxi, China; ^3^ Institute of Medical Research, Northwestern Polytechnical University, Xi’an, Shaanxi, China; ^4^ Department of Histology and Developmental Biology, Tianjin Medical University, Tianjin, China; ^5^ Tianjin Key Laboratory of Ocular Trauma, Laboratory of Molecular Ophthalmology, Tianjin Medical University, Tianjin, China; ^6^ School of Medicine, Nankai University, Tianjin, China

**Keywords:** traumatic proliferative vitreoretinopathy, inflammation, Olink, biomarkers, interleukin, chemokine

## Abstract

**Background:**

The aim of this study was to identify inflammatory biomarkers in traumatic proliferative vitreoretinopathy (TPVR) patients and further validate the expression curve of particular biomarkers in the rabbit TPVR model.

**Methods:**

The Olink Inflammation Panel was used to compare the differentially expressed proteins (DEPs) in the vitreous of TPVR patients 7–14 days after open globe injury (OGI) (*N* = 19) and macular hole patients (*N* = 22), followed by correlation analysis between DEPs and clinical signs, protein–protein interaction (PPI) analysis, area under the receiver operating characteristic curve (AUC) analysis, and function enrichment analysis. A TPVR rabbit model was established and expression levels of candidate interleukin family members (IL-6, IL-7, and IL-33) were measured by enzyme-linked immunosorbent assay (ELISA) at 0, 1, 3, 7, 10, 14, and 28 days after OGI.

**Results:**

Forty-eight DEPs were detected between the two groups. Correlation analysis showed that CXCL5, EN-RAGE, IL-7, ADA, CD5, CCL25, CASP8, TWEAK, and IL-33 were significantly correlated with clinical signs including ocular wound characteristics, PVR scoring, PVR recurrence, and final visual acuity (*R* = 0.467–0.699, *p* < 0.05), and all with optimal AUC values (0.7344–1). Correlations between DEP analysis and PPI analysis further verified that IL-6, IL-7, IL-8, IL-33, HGF, and CXCL5 were highly interactive (combined score: 0.669–0.983). These DEPs were enriched in novel pathways such as cancer signaling pathway (*N* = 14, *p* < 0.000). Vitreous levels of IL-6, IL-7, and IL-33 in the rabbit TPVR model displayed consistency with the trend in Olink data, all exhibiting marked differential expression 1 day following the OGI.

**Conclusion:**

IL-7, IL-33, EN-RAGE, TWEAK, CXCL5, and CD5 may be potential biomarkers for TPVR pathogenesis and prognosis, and early post-injury may be an ideal time for TPVR intervention targeting interleukin family biomarkers.

## 1 Introduction

Proliferative vitreoretinopathy (PVR) is characterized by the formation and contraction of fibrotic membranes on the retinal surface and involves three overlapping phases: inflammation, proliferation, and remodeling. It is known that multiple intraocular cell types, cytokines, growth factors, chemokines, matrix proteins, and multiple signaling pathways interact to promote this complex pathological process. A large number of cytokines have been reported to be involved in the pathogenesis of PVR, including PDGF, VEGF, HGF, EGF, TGFα/β, FGF, IL-1, IL-6, IL-8, IL-10, TNF-α/β, IFN-γ1, and GFAP (1–7). However, different immune responses and mechanisms may be active in the pathogenesis of PVR and traumatic proliferative vitreoretinopathy (TPVR) (8, 9), and when it comes to TPVR-related cytokines, only IL-6, IL-8, ABCA4, TNF-α, and HGF have been mentioned in relevant reports (8, 10, 11).

Prevention and intervention of TPVR are two important aspects of this challenging task. The standard practice worldwide for OGI-PVR is to conduct primary surgical repair at the earliest opportunity to preserve the structural integrity of the globe (12). Vitrectomy is often undertaken 7–14 days thereafter to reconstruct the intraocular tissues; however, the outcomes are frequently unsatisfactory and there are also no effective medical treatments available yet. The use of biomarkers in the detection and management of disease has become an important tool in modern clinical medicine, and their application to TPVR should be no exception. The strong inflammatory response immediately after OGI is the initiating factor for the formation of TPVR, and it is of great importance to identify effective inflammation-related biomarkers during this process, which may provide an important reference for future drug use. However, the multiple cytokines involved in the onset and development of this disease make targeted treatment challenging, and targeting single cytokine is always ineffective.

**Figure 1:**
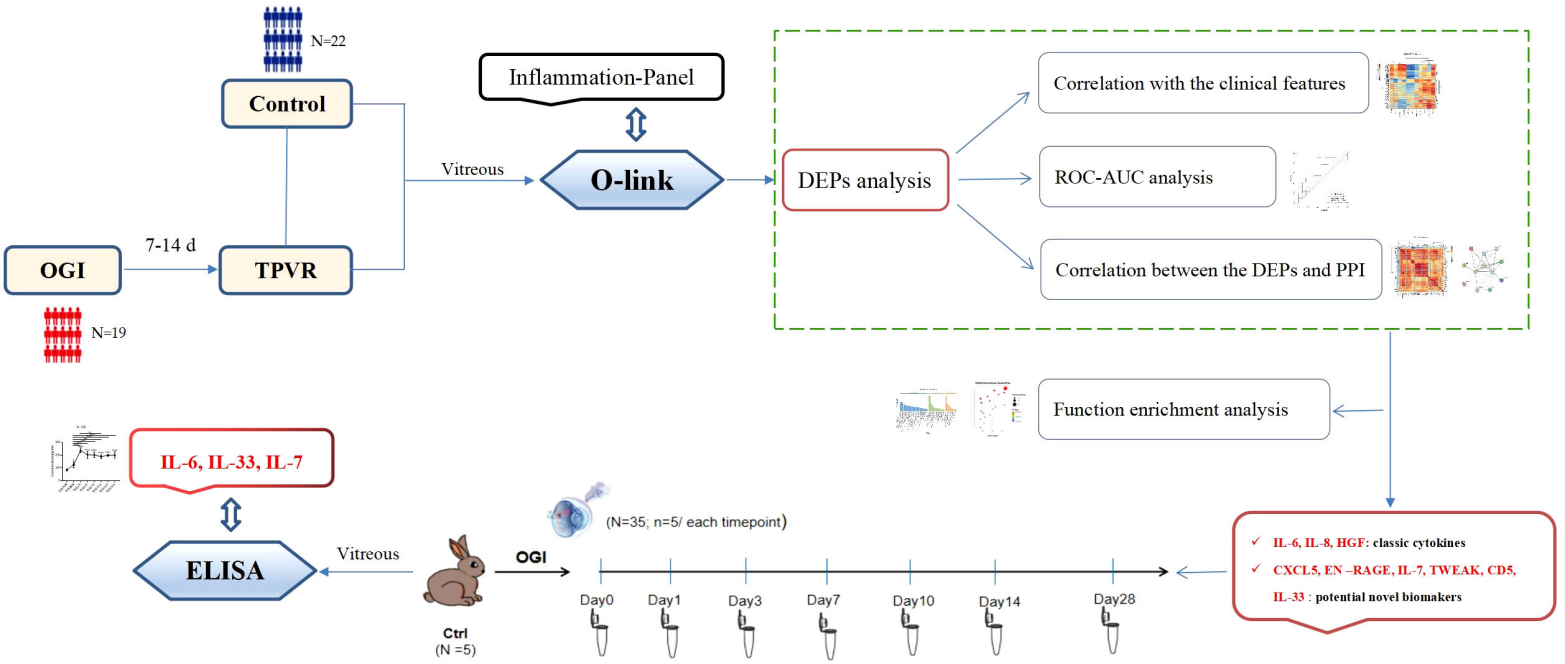
Overview of the study. OGI: open globe injury, TPVR: traumatic proliferative vitreoretinopathy, DEPs: different expression proteins, ROC: receiver operating characteristic, AUC: the area under the receiver operating characteristic curve, PPI: protein–protein interaction, ELISA: enzyme-linked immunosorbent assay.

With the recent advent of Olink proteomic technologies, it is now possible to simultaneously detect the expression levels of 92 cytokines with only a small amount of sample, and the excellent reproducibility and stability in detection of plasma samples have been demonstrated in previous studies (13, 14). Therefore, it is an ideal option for the TPVR study, providing a more comprehensive analysis of the proteins involved in this disease. This study uses an inflammatory panel to identify inflammatory biomarkers associated with TPVR, while providing a preliminary proteomic reference for further investigation. We select some valuable and novel biomarkers and explore their role and possible mechanism in the development of TPVR through functional enrichment analysis. Furthermore, we validate the expression curve of selected biomarkers during 28 days after injury using the rabbit TPVR model, thus providing evidence for optimal targeted intervention timing, which was difficult to achieve due to the limited schedule of vitrectomy in clinical practice (**Figure 1**).

## 2 Materials and methods

### 2.1 Participants

The study protocol adhered to tenets of the Declaration of Helsinki and was approved by the institutional ethics committee of Tianjin Medical University General Hospital. All participants gave written informed consent for use of their intraocular specimen and clinical records. Nineteen patients (19 eyes) with OGI who developed PVR and required vitrectomy were prospectively recruited from Tianjin Medical University General Hospital from February 2021 to June 2022. For intraocular foreign bodies and traumatic endophthalmitis, emergency vitrectomy was required in all cases if necessary when PVR had not formed. Moreover, the presence of microbial infectious inflammation in the two groups was not identical to the aseptic inflammation that occurs during the formation of PVR, and for the sake of relative consistency of the samples included in the study, such cases with the possibility of infection were excluded. Moreover, patients with closed globe injury, previous ocular anomalies, a history of ocular trauma and intraocular surgery, and diabetes; those who are younger than 18 years; and those with severe comorbidities were also excluded. Twenty-two patients (22 eyes) who underwent vitrectomy for macular hole during the same period were included as controls. A total of 41 vitreous samples were collected for Olink proteomics analysis. Clinical characteristics of the participants were carefully recorded, including age, cause of injury, location of injury, initial visual acuity, type of injury (NOI), time between injury and emergency surgery (TIES), interval between injury and vitrectomy (IOIV), total wound length (TWL), distance from the limbus to the most posterior full thickness scleral wound (DLP), scleral wound length (SWL), retinal retention (Retinal-R) and PVR score at vitrectomy (PVR-S), PVR recurrence after vitrectomy (PVR-R), and best-corrected visual acuity in Log-MAR (BCVA-L) at the last follow-up. The TPVR scoring system we used has been described previously (15) (**Supplementary files**, **Supplementary Table 1**).

### 2.2 Vitreous collection in TPVR patients

Vitreous was obtained at the beginning of vitrectomy using a 23G vitreous cutter, immediately placed on ice and transferred to sterile tubes, centrifuged at 4°C for 10 min to remove blood cells or other confounding cellular material, then transferred to another sterile tube and frozen at −80°C for immediate future use.

### 2.3 Protein extraction and cytokine measurement

Vitreous samples were measured using the Olink^®^ INFLAMMATION panel* (Olink Proteomics AB, Uppsala, Sweden) according to the manufacturer’s instructions, which allows the simultaneous analysis of 92 analytes using 1 µL of each sample (13, 14, 16). Detailed information about the panel can be found in the **Supplementary files**, **Supplementary Data Sheet 1**. The final assay result is presented as Normalized Protein Expression (NPX) values, which is an arbitrary unit on a log2 scale and a higher value corresponds to higher protein expression. However, NPX values cannot be compared among diverse proteins.

**Figure 2:**
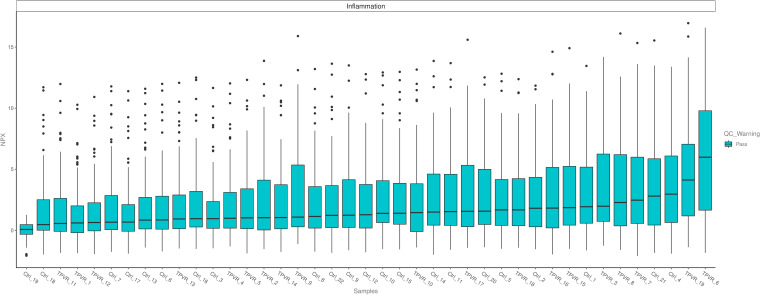
The quality control testing of all samples.

Quality control (QC) of the data is performed in two steps as previously described (14). All assay validation data (detection limits, intra- and inter-assay precision data, etc.) are available on the manufacturer’s website (www.olink.com). All samples were successfully measured and passed QC in the current study (**Figure 2**).

### 2.4 Potential biomarkers’ screening

The R package Olink ^®^Analyze was used to identify sets of differentially expressed proteins (DEPs) between the two groups. The correlation between DEPs and clinical signs in TPVR patients was determined, and the clustering correlation heatmap with signs was performed using OmicStudio tools at https://www.omicstudio.cn. The clinical parameters analyzed were age, TIES, NOI, IOIV, TWL, DLP, SWL, Retinal-R, PVR-S, PVR-R, and final BCVA-L. The correlation between the expression levels of two DEPs was determined, and the clustering correlation heatmap with signs was performed using the OmicStudio tools at https://www.omicstudio.cn. A protein–protein interaction (PPI) network was constructed using the STRING database to further visualize the interaction of all DEPs and the candidate biomarkers. The diagnostic performance of the DEPs was assessed by receiver operating characteristic (ROC) curves generated using the ROCR package, with the higher the areas under the ROC (AUC) value reflecting better classifier performance. Only those that were found to be highly significant in all of the above bioinformatic analyses were considered as potential biomarkers for TPVR.

### 2.5 Function enrichment analysis

Gene Ontology (GO) and Kyoto Encyclopedia of Genes and Genomes (KEGG) enrichment analyses were also performed using ggplot2. All significant DEPs were mapped to each term or pathway of the GO or KEGG database, and the GO term or KEGG pathway that was significantly enriched in DEPs compared to the whole genome background was identified using a hypergeometric test.

### 2.6 Rabbit TPVR model establishment and vitreous collection

**Figure 3:**
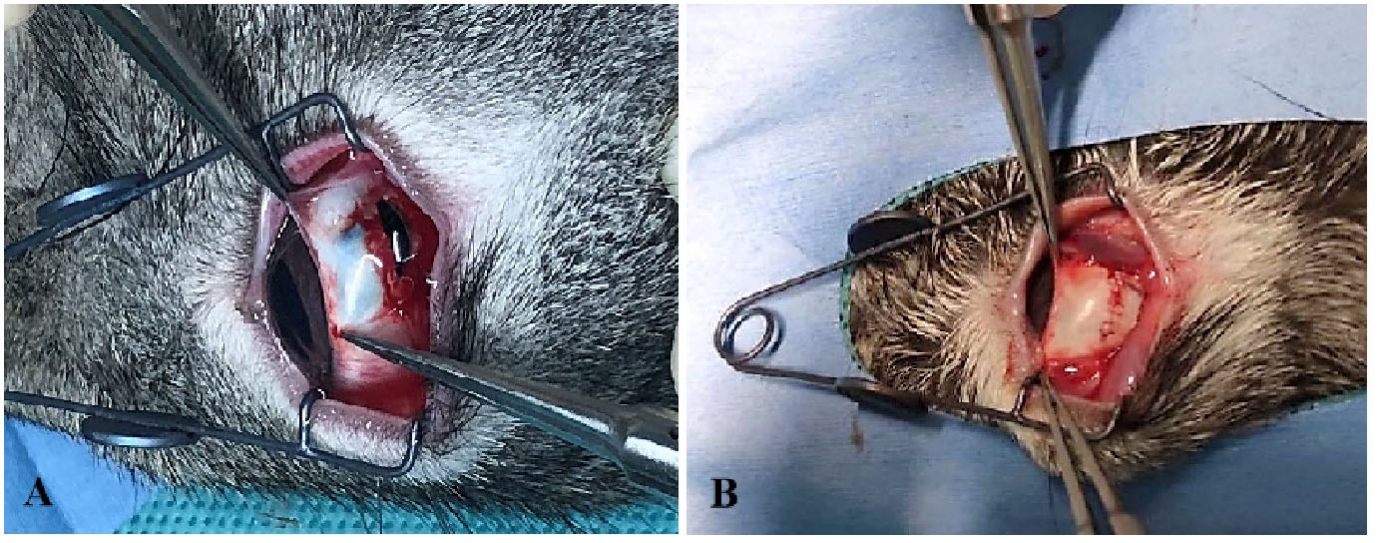
The establishment of the rabbit TPVR model. **(A)** A 6-mm full-thickness scleral wound was made; **(B)** the wound was sutured 4 h after injury.

Rabbits were treated according to the Association for Research in Vision and Ophthalmology animal statement and housed in the Laboratory of Experimental Animals, Tianjin Medical University General Hospital. Healthy Belgian rabbits with no pre-existing fundus abnormalities were selected and the rabbit TPVR model was implemented as described previously with slight modifications (17) (**Figure 3**). Briefly, a 6-mm penetrating wound circumferentially 5.0 mm behind the limbus at the supratemporal quadrant of the right eye was made, and the wound was sutured 4 h later with an aseptic technique, followed by intravitreal injection of 0.1 mL of autologous serum. Blood was drawn via the ear vein immediately before surgery. B-scan ultrasonography and hematoxylin–eosin (HE) staining were used to confirm the establishment of the model.

Approximately 0.5 mL of vitreous was collected at baseline, 1, 3, 7, 10, 14, and 28 days after OGI using 24G syringes (*n* = 5 rabbits for each time point). Normal rabbit vitreous was collected as control (*n* = 5). Vitreous was collected only once for each rabbit. Concentrations of IL-6, IL-7, and IL-33 were measured using enzyme-linked immunosorbent assay (ELISA) kits (Jiangsu Jingmei Biological Technology Co., Cheng, China) according to the manufacturer’s instructions.

### 2.7 Statistical analysis

All statistical analyses of the Olink test were performed using the R software “Olink^®^Analyze” (R version 3.6.3). For the ELISA test, the different groups were compared using ANOVA. *p* < 0.05 was considered statistically significant.

## 3 Results

### 3.1 Clinical characteristics of the participants

**Tables 1A**, **B** shows the clinical characteristics of all the participants in the Olink study. A total of 41 patients were included in this cohort.

**Table 1A:** Clinical features of TPVR (*N* = 19).

Sex: Male/Female	14/5
Age (years)	53.8 (23–74)
Cause of injury Assault Traffic accident Fall down	14 2 3
Place of injury Workplace Home Road Public places	8 6 2 3
Initial VA (Log MAR)	2.74 (2.5–3.0)
Nature of injury Laceration Globe rupture	4 15
TWL (mm)	20.2 (5–36)
SWL (mm)	18.5 (5–36)
DLP (mm)	9.2 (1–25)
Zone 1, 2, and 3 1, 2 2, 3 2 only 3 only	2 1 9 3 4
TIES (h)	22.7 (5–96)
PVR-S	7.4 (1–14)
IOIV (days)	10.2 (7–14)
Retinal retention (%)	80 (5–100)
Final BCVA (Log-MAR)	1.8 (0.1–3)
PVR recurrence	57.9%
Retinal attachment rate	86.8%

**Table 1B:** Clinical features of control group (*N* = 22).

Diagnosis	Macular Hole
Number	22
Age (years)	65.9 ± 6.5
Gender (M:F)	5:17
Pre-operative VA (logMAR)	1.0 ± 0.3
Duration of symptoms (weeks)	17.8 ± 18.1

### 3.2 Potential biomarkers screening

**Figure 4:**
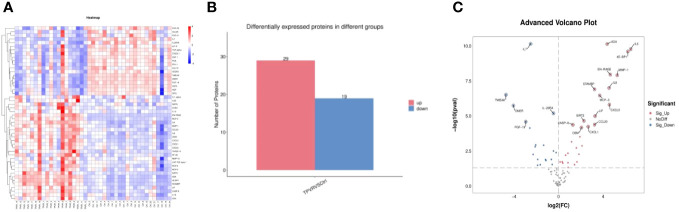
All differentially expressed inflammation-related biomarkers between TPVR and control group. **(A)** Heat map of 48 DEPs; **(B)** 29 proteins upregulated, 19 proteins downregulated; **(C)** volcanic visualization of 92 inflammation-related biomarkers. Red, significantly upregulated proteins; gray, no differences; blue, significantly downregulated proteins.

**Figure 5:**
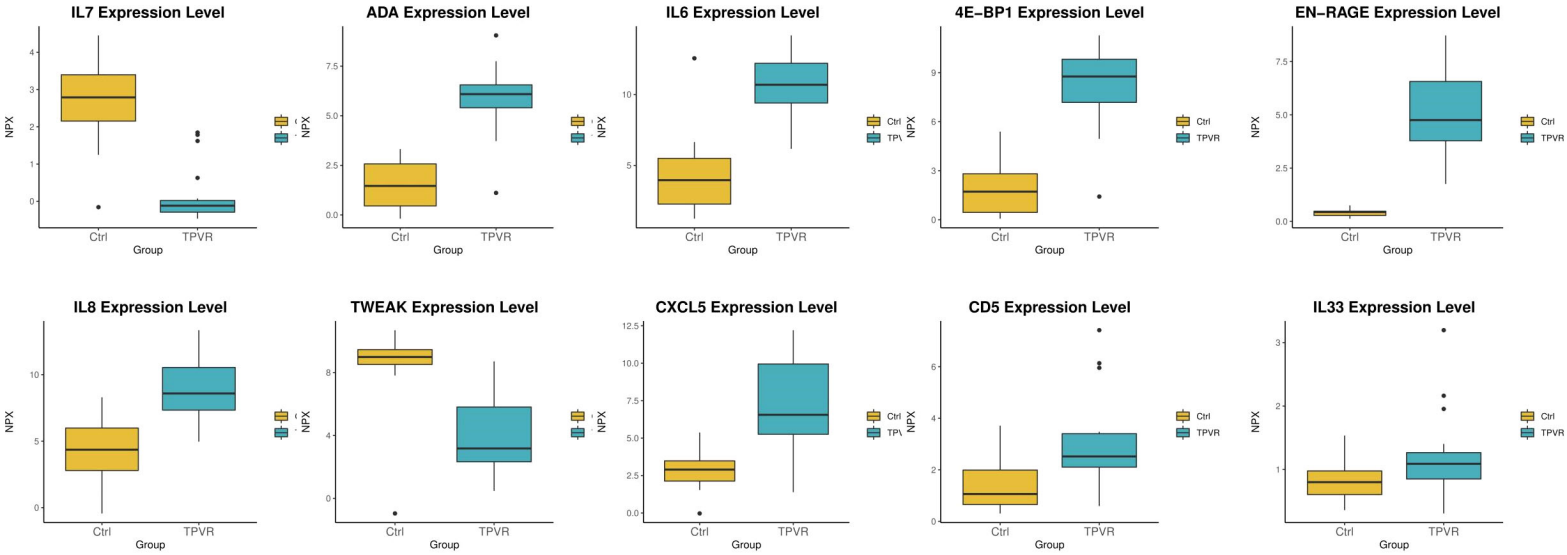
Box scatter plot of the five DEPs (4E-BP1, ADA, EN-RAGE, IL-6, and IL-7) with the largest fold change and five other potential biomarkers (CXCL5, IL-33, CD5, TWEAK, and IL-8).

Compared to the control group, 48 DEPs were identified in the TPVR group, of which 29 were upregulated and 19 were downregulated (**Figure 4**), mainly covering members of the chemokine, interleukin, and growth factor families. The expression heatmap of the above DEPs in each sample is shown in **Figure 4A**. The top 5 DEPs according to the smallest *p*-value were 4E-BP, ADA, EN-RAGE, IL-6, and IL-7 (**Figure 5**). Detailed information can be found in the **Supplementary file** (**Supplementary Table 2**).

**Figure 6:**
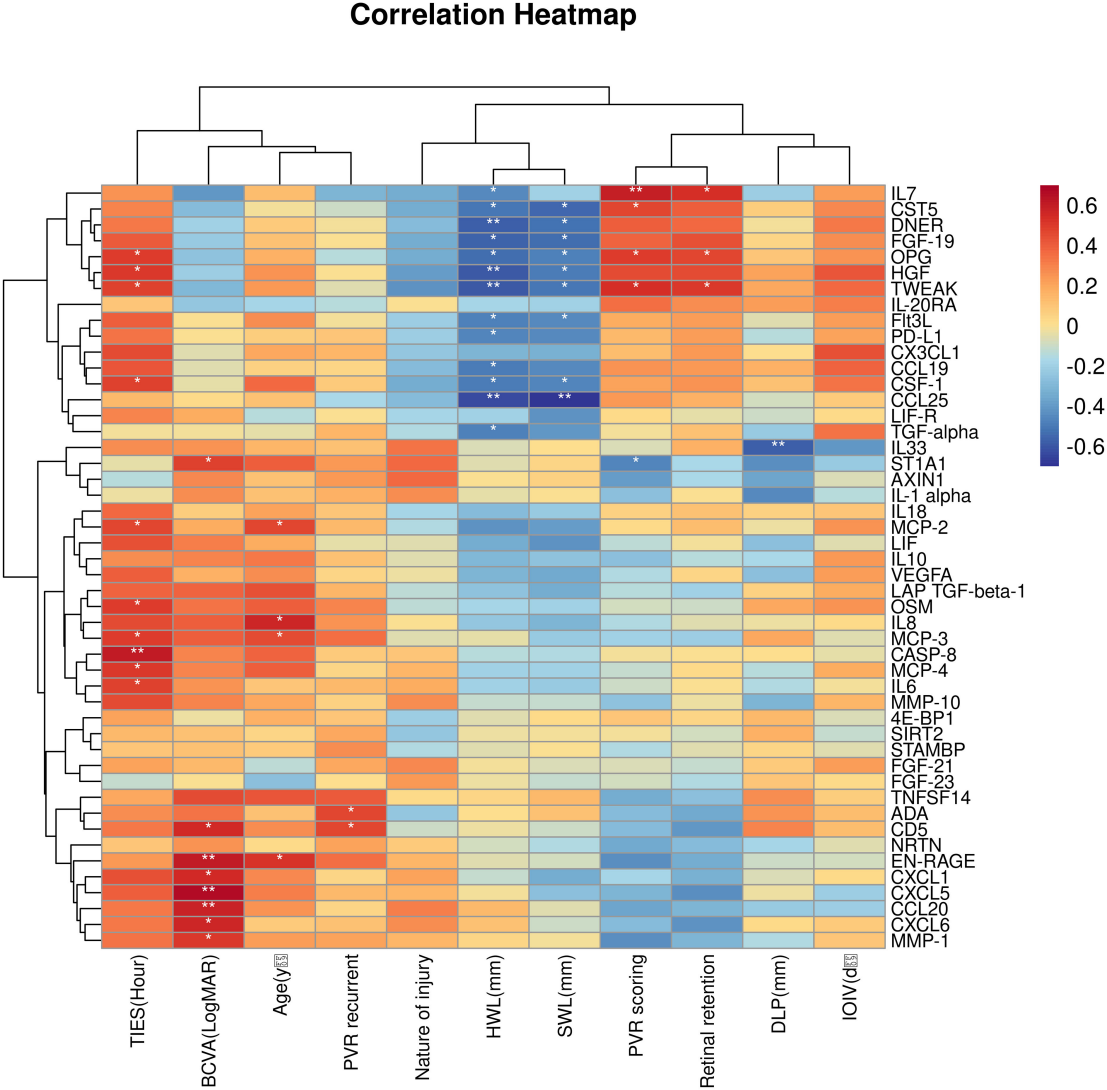
Correlation heatmap between the DEPs and clinical features. Orange, positively related; blue, negatively related; and white, nonrelated. *p < 0.05, **p < 0.01.

The association between the 48 DEPs and clinical characteristics of TPVR patients was analyzed, and 29 DEPs were found to be correlated (**Supplementary files**, **Supplementary Table 3**, **Figure 6**). CXCL5, EN-RAGE, and CD5 were positively correlated with final BCVA-L. IL-7 was positively correlated with PVR-S. ADA and CD5 were positively correlated with PVR-R. Massive cytokines were correlated with TIES (especially IL-6 and CASP8), TWL (especially CCL25 and TWEAK), SWL (especially CCL25), and DLP (especially IL-33). However, none was correlated with IOIV and NOI. Based on the above analysis, we primarily considered IL-6, IL-7, CXCL5, EN-RAGE, CASP8, TWEAK, IL-33, CCL25, ADA, and CD5 as candidate biomarkers for further investigation.

**Figure 7:**
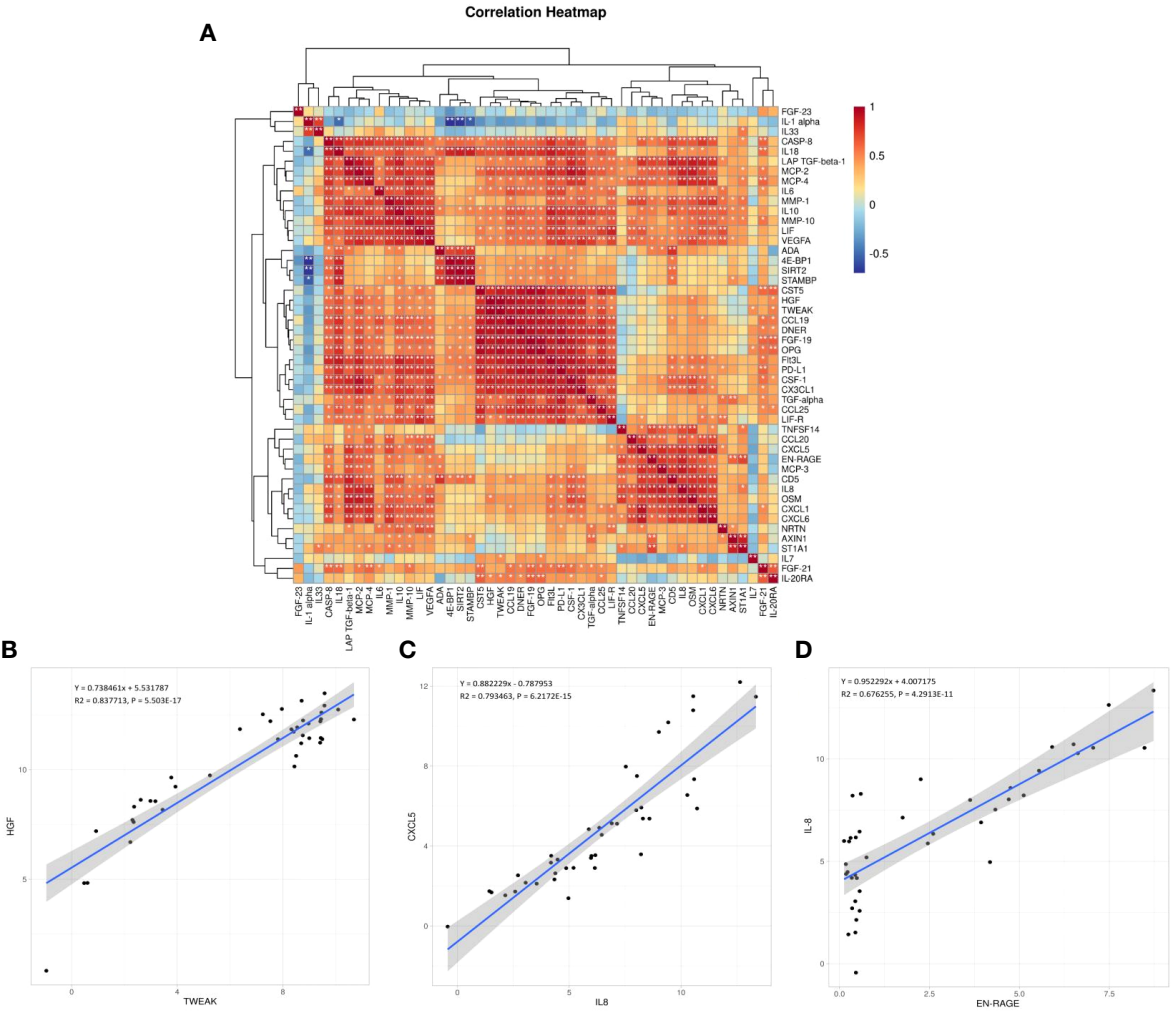
**(A)** Correlation heatmap between the DEPs. Orange, positively related; blue, negatively related; and white, nonrelated.*p < 0.05, **p < 0.01, ***p < 0.001, ****p < 0.0001. **(B–D)** The scatter plots are visualizations of these three pairs of inflammation-related biomarkers: TWEAK and HGF (the most significant positive correlation, B), CXCL5 and IL-8 **(C)**, EN-RAGE and IL-8 **(D)**; *R*: Pearson correlation coefficient.

**Figure 8:**
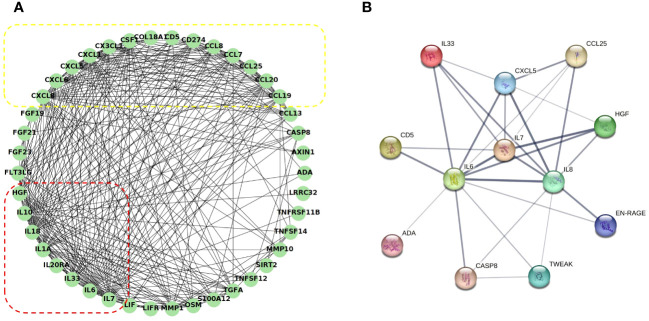
Co-expression network and identification of hub proteins: **(A)** The PPI network between all the DEPs was established by using the STRING database. The node represents the protein, and the edge represents the relationship between the proteins; dashed red box: interleukin family members highly interactive; dashed yellow box: chemokine family members highly interactive. **(B)** The PPI network between the potential biomarkers was established by using the STRING database. The edge width represents the combined score. PPI, protein–protein interaction.

The correlation between the 48 DEPs is shown in the correlation heatmap in **Figures 7A, B**. TWEAK and HGF had the most significant positive correlation, and 4E-BP and IL-1α had the most significant negative correlation. CXCL5 and EN-RAGE were also highly correlated with IL-8 (**Figure 7C, D**); detailed information can be found in the **Supplementary files** (**Supplementary Table 4**, *R* > 0.65 would receive more attention). Chemokine and interleukin family members were highly activated and strongly interconnected in the PPI analysis (**Figure 8A**). A PPI network for certain DEPs was also created to further explore the connection of these candidate proteins, and the proteins included are the top 5 proteins with the highest fold change and the candidate biomarkers mentioned above. IL-6, IL-8, IL-7, HGF, CXCL5, CCL25, and IL-33 were all strongly interconnected in the candidate biomarkers’ PPI analysis (**Figure 8B**). IL-6 appeared to be a central hub protein as it was connected to all the other 11 proteins, and it was most strongly connected to IL-8 (combined score of 0.991). In addition, IL-7 was most strongly connected with HGF (combined score of 0.991).

**Figure 9:**
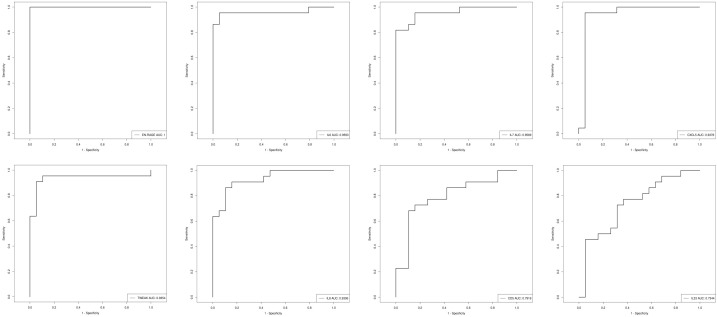
The AUC curve of EN-RAGE, IL-6, IL-7, CXCL5,TWEAK, IL-8, CD5, and IL-33; the AUC value is shown in the figure legend at the bottom right corner.

ROC analysis was performed and the results showed that all the above candidate biomarkers had high AUC values (0.734–1), of which EN-RAGE with the highest AUC generated 100% sensitivity and 5% specificity. **Figure 9** shows the AUC curve of some candidate biomarkers. The AUC values for all DEPs are listed in **Supplementary files** (**Supplementary Table 2**).

IL-6, IL-7, IL-8, IL-33, CXCL5, EN-RAGE, TWEAK, and CD5 may be potential biomarkers for TPVR pathogenesis and prognosis based on all the aforementioned bioinformatics analyses.

### 3.3 Function enrichment of DEPs

**Figure 10:**
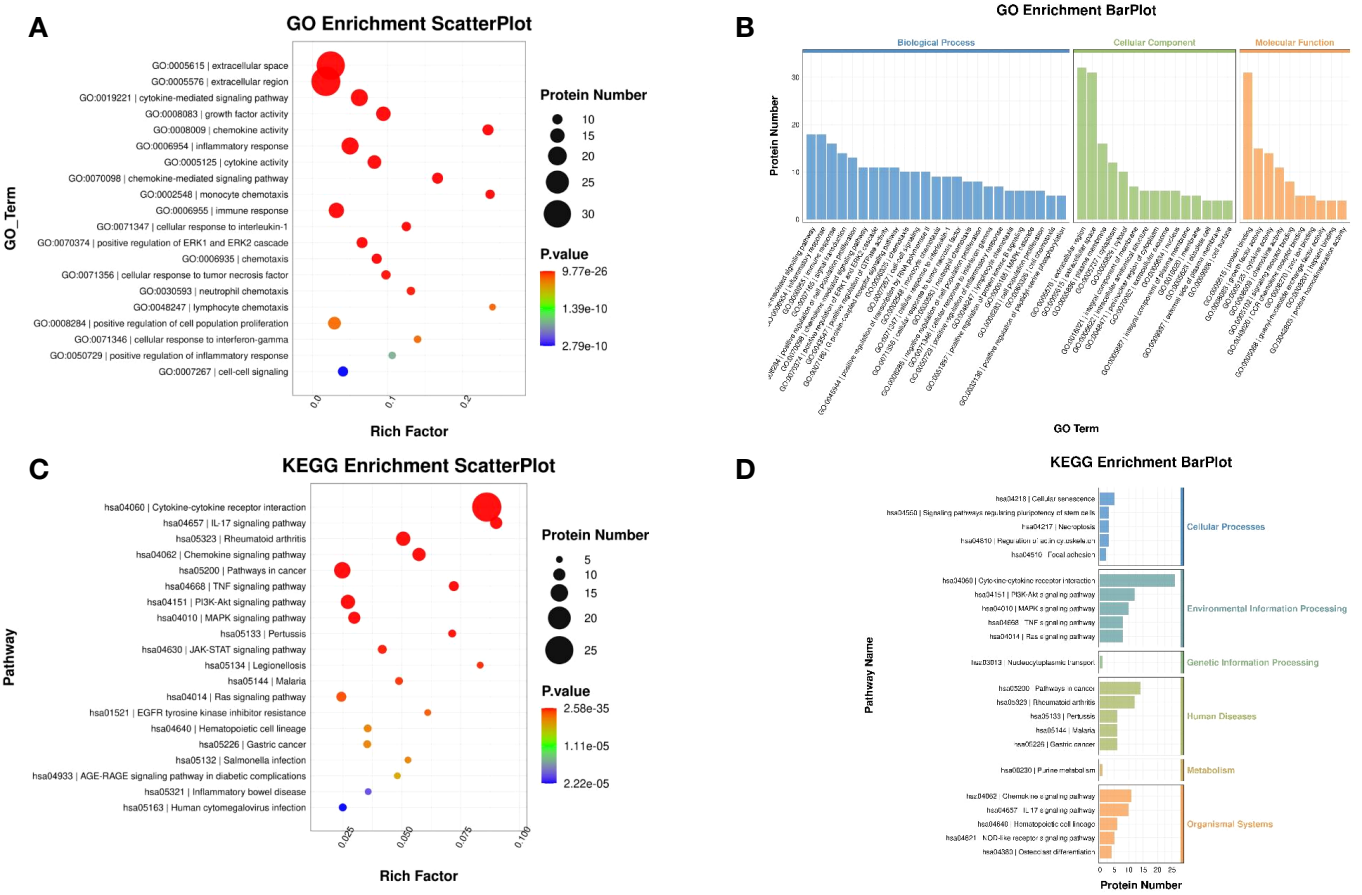
GO and KEGG enrichment analysis of DEPs. **(A)** Scatter plot of GO: The color of the bubbles indicates the significance of the enriched GO terms. **(B)** Bar plot of GO: The abscissa indicates the enrichment by GO functional classification and the ordinate represents the number of enriched proteins. **(C)** Scatter plot of KEGG: The color of bubbles indicates the significance of the enriched KEGG pathway. **(D)** Bar plot of KEGG: The ordinate represents the significantly enriched KEGG pathways and the abscissa represents the number of proteins enriched in each KEGG pathway (rich factor ≤ 1).

To further investigate the potential function of the DEPs, GO and KEGG enrichment analysis was conducted (**Supplementary files**, **Supplementary Tables 5 and 6**). The results of GO analysis showed that the genes of DEPs in biological processes were mainly enriched in cytokine-mediated signaling pathway and inflammatory response. Cellular component was mainly enriched in extracellular space and extracellular region. The molecular function category was mainly enriched in growth factor activity and chemokine activity (**Figures 10A, B**). KEGG analysis showed that the functions of the DEPs were mainly focused on cytokine–cytokine receptor interaction, rheumatoid arthritis, and pathways in cancer (**Figures 10C, D**).

### 3.4 Validation of potential biomarkers in the TPVR rabbit model

**Figure 11:**
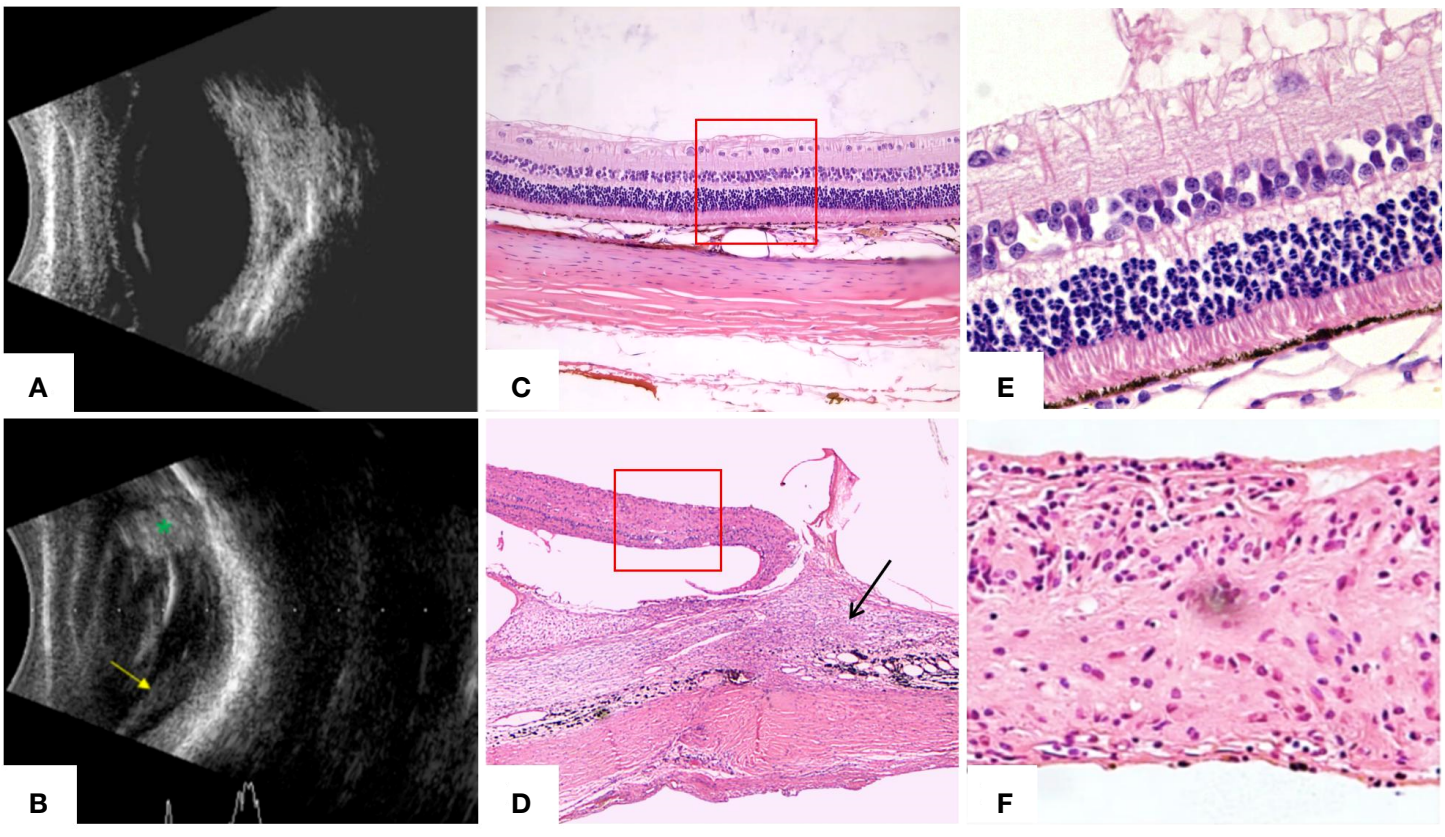
B-scanning and HE staining confirmed the establishment of the rabbit traumatic PVR model at 28 days. **(A)** Ultrasound of normal rabbit eyes showed flattened retina and no abnormal echoes in the vitreous cavity. **(B)** Ultrasound of rabbit eyes in the TPVR model showed a large number of dots and bands of echoes in the vitreous cavity, localized retinal detachment (yellow arrowheads), and enhanced echoes at the wound site (green *). **(C)** HE staining of the structure of each layer of the retina in normal rabbit eyes (×40). **(D)** HE staining of the TPVR showed localized injury as large areas of subretinal proliferative membranes, vitreoretinal traction, retinal detachment, and folding and curling to the contralateral side (black arrow is the proliferation and the red solid box is the detached retina) (×40). **(E)** HE staining of each layer of the structure of the retina in normal rabbit eyes (×100). **(F)** HE staining of the local retinal structure within the red box in **(D)** shows that there is a disruption in the structure of the retina, with the structural arrangement of the layers in disarray and the absence of multilayers of cells (×100).

**Figure 12:**
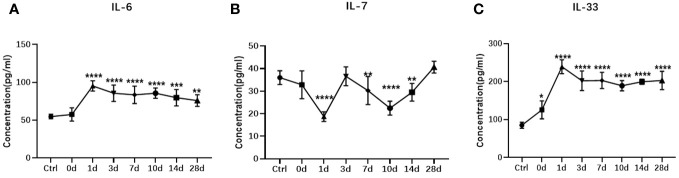
ELISA measurement of vitreous IL-6 **(A)** , IL-7 **(B)**, and IL-33 **(C)** in the rabbit TPVR model. N = 5 animals/time point. * represent data compared with Ctrl, *p < 0.05, **p < 0.01, ***p < 0.001, ****p < 0.0001.

Intense inflammation typically occurs after ocular trauma. Therefore, we further carried out ELISA verification for the interleukin family members of chosen biomarkers. B-scanning and HE staining confirmed the establishment of the rabbit TPVR model (**Figure 11**). Expression levels of IL-6 was significantly upregulated, peaking 1 day after injury, and remained at high levels until 28 days after injury (**Figure 12A**). IL-7 was significantly downregulated in 1, 7, 10, and 14 days after injury (**Figure 12B**). IL-33 was significantly upregulated, peaking 1 day after injury, and remained at high levels until 10 days after injury (**Figure 12C**). All are consistent with the trend observed in Olink data (**Figure 12**, **Supplementary files**, **Supplementary Table 7**).

## 4 Discussion

Although the pathological mechanism is very complex, it is widely accepted that inflammation is central to the onset and development of TPVR, and a key factor in optimizing therapeutic management is the identification of sensitive and reliable inflammatory protein biomarkers. In the present study, we used Olink to identify protein differences in TPVR patients by inflammation panel. The 92 protein biomarkers covered pro- and anti-inflammatory cytokines, chemokines, growth factors, and factors involved in acute inflammatory and immune responses, angiogenesis, fibrosis, and endothelial activation. Forty-eight DEPs were identified, indicating an excessive inflammatory response in the TPVR process at the molecular level for the first time. For ethical reasons, it is impossible to obtain fresh human vitreous samples from healthy eyes. Therefore, we chose macular hole patients as control. The complex GO and KEGG enrichment also indicated that TPVR pathogenesis is a sophisticated biological process and multifaceted mechanism involved. In addition to traditional biomarkers such as IL-6 and IL-8, we also identified some novel biomarkers through a series of bioinformatic analyses. In addition to previously identified pathways such as NF-κB, JAK/STAT, and TGF-β (1, 2), novel pathways such as cytokine–cytokine receptor interaction pathways were also highly enriched. Candidate biomarkers such as IL-7, TWEAK, 4E-BP, CXCL5, and CD5 are all actively involved in cancer initiation and progression in previous reports (18–22), and the KEGG analysis also indicated cancer pathway enrichment. This may be due to the similar biological processes of these two diseases, such as overgrowth, proliferation, and epithelial-to-mesenchymal transition (EMT). PPI analysis of all DEPs suggested that interleukin and chemokine families are actively involved in TPVR and strongly interact. IL-6, IL-8, IL-7, IL-33, HGF, and CXCL5 were all strongly interconnected and highly expressed in TPVR by the PPI network for certain DEPs, suggesting that they may work together mechanistically, and IL-6 appeared as the central hub protein, indicating its core role in TPVR.

Among the selected biomarkers, IL-6, IL-7, and IL-33 all belong to the interleukin family. IL-6 is a potent pro-inflammatory cytokine that significantly upregulated in many inflammation-related diseases and also exhibits a wide range of biological activities such as stimulating immune responses, stimulating glial and fibroblast proliferation, and promoting collagen synthesis during wound healing after trauma, especially burns or other tissue damage leading to inflammation (23). IL-6 expression was upregulated in previous PVR studies (7, 23–27), especially in TPVR (8) and has been recommended as an important target for PVR treatment (23–27). In accordance with a previous study (8), IL-6 was significantly elevated in the TPVR group with particular high diagnostic value in our study. The PPI analysis also showed its core role in TPVR. Previous research has demonstrated a positive correlation between IL-6 levels and the extent and duration of PVR grade (23). However, in our study, there was no correlation between IL-6 levels and PVR-S, but it was positively correlated with TIES, suggesting that completing emergency surgery as soon as possible may alleviate the subsequent inflammatory response. IL-7 plays a critical role in the development, proliferation, and differentiation of T cells and the maturation of B cells through activation of IL-7R (19, 28). It can reconstitute the immune system, enhance its function, and antagonize the immunosuppressive network (29, 30). Previous studies have shown that IL-7 plays an important role in wound healing by inhibiting TGF-β production in fibroblasts (31–34). Kim SY reported that IL-7 plays a critical role in corneal fibroblasts in granular corneal dystrophy type 2 by reducing the expression of TGF-β and TGFBI through regulation of the RNNKI/RANK signaling cascade and by regulating the expression of MT-MMP (35). Here, we identified a potential role of IL-7 in TPVR patients for the first time, with the largest fold change among all DEPs and a high AUC value of 0.96. Since TGF-β plays a key role in PVR, this suggests that IL-7 may affect PVR by activating the TGF-β pathway. Moreover, IL-7 was positively correlated with PVR-S, meaning that IL-7 may ameliorate the PVR severity to some extent, which may facilitate the manipulation of vitrectomy, consistent with previous studies showing that IL-7 slows wound healing (31). The potential protective role of IL-7 and its mechanism in TPVR is still a matter for further investigation. Among these DEPs, IL-33 caught our attention, which belongs to IL-1 family and is famous for its alarmin signal after injury and associated with the sterile inflammation caused by mechanical trauma. It is considered to be a dual-function protein, exerting its pro- or anti-inflammatory effects (36), with pro-inflammatory roles in retinal and retinal pigment epithelial (RPE) cells (37–39). Previous clinical studies have shown that serum IL-33 levels rise sharply immediately after injury, and initial IL-33 levels may serve as an indicator of impending death in polytraumatized patients (40). The Ricker LJ study showed no significant changes in IL-33 in patients with recurrent PVR after rhegmatogenous retinal detachment (RRD) compared to those without postoperative PVR (41). However, IL-33 was upregulated in TPVR patients in our study and its correlation with DLP also suggests its association with trauma severity. Müller glial cells are the primary source of IL-33 in the retina (37, 42) and it is significantly increased after RPE cell activation and stimulates further release of inflammatory factors such as IL-6, IL-8, and TNFα, which regulate tissue remodeling (43). IL-33 is also known for its profibrotic function and increases retinal fibrosis after laser injury (39, 44), all of which provide some clues as to the mechanism for its involvement in TPVR that require further in-depth investigation.

Chemokines, as an important component of inflammatory factors, have attracted the attention of some scholars in the development of PVR. IL-8, also CXCL1, is mainly secreted by RPE cells, macrophages, and endothelial cells, with HRPE cells being the major source (8, 45, 46). It has angiogenic and fibrocellular proliferative effects on ocular tissues (8, 45, 46) and is particularly important in acute inflammation. The increased levels of IL-8 in the vitreous of the TPVR groups in the present study were in agreement with the previous study by Canataroglu H (8), which showed that IL-8 levels were elevated in all (100%) of the traumatic PVR patients, combined with the high AUC value of 0.93, all suggesting that IL-8 may be an important mediator in the pathogenesis of TPVR. Ayako Yoshida (46) reported that mechanical injury triggers HRPE IL-8 secretion, which may lead to neutrophil recruitment in the retinal wound healing response, shedding light on the mechanism of IL-8 in TPVR. A significant positive correlation between IL-8 and CXCL5 was found here, which is consistent with the previous study that CXCL5 is homologous to IL-8 (47). CXCL5 is released by inflammatory cells through activation of IL-1 or TNFα, interacts with CXCR2, has a strong chemotactic effect on neutrophils, is particularly important for leukocyte infiltration in inflammatory diseases, and is also involved in angiogenesis and connective tissue remodeling (48, 49). CXCL5/ENA78 is also responsible for several cellular functions, including proliferation, migration, and EMT, which are important steps in PVR formation (18, 50). Zandi S et al. reported that CXCL5 was significantly upregulated in patients with primary RRD who developed postoperative PVR compared to eyes without postoperative PVR, suggesting its potential predictive value for the development of postoperative PVR (51). In this study, CXCL5 expression was significantly increased in TPVR patients and correlated with final BCVA-L, implying that inhibition of CXCL5 is an attractive target for improving TPVR prognosis.

TWEAK is a TNFSF member that activates the fibroblast growth factor inducible-14 (Fn14) or CD163 receptor to trigger multiple signaling cascades leading to a variety of cellular events including proliferation, migration, differentiation, apoptosis, angiogenesis, and inflammation. The function of TWEAK varies depending on the receptor to which it binds, the tissue type in which it is expressed, specific extrinsic conditions, and the presence of other cytokines. It is protective in healthy tissues, but detrimental in chronic inflammatory conditions (52, 53). Previous studies have identified a potential role for TWEAK/Fn14 in pro-fibrosis in various organs or tissues (52–58). Chen DY reported TWEAK in proliferative diabetic retinopathy (PDR) and promoted proliferation and collagen synthesis in retinal ARPE-19 cells (59). In our study, the upregulation of TWEAK in TPVR patients, the particularly high AUC value, the most significant positive correlation with HGF, and the correlation with PVR-S led us to propose TWEAK as another candidate target biomarker for the diagnosis of TPVR and for the assessment of PVR severity.

The main challenge of TPVR is recurrence and poor prognosis, which deserves more attention. In the present study, CD5 expression levels were correlated with PVR-R and BCVA-L, and EN-RAGE was correlated with final BCVA-L, suggesting that they may be potential biomarkers for the prognosis of TPVR. CD5 is a transmembrane glycoprotein that regulates T-cell function and development and is expressed on the surface of dendritic cells. The presence of CD5+ DCs has been correlated with increased patient survival in a variety of tumors (60). He et al. (61) identified CD5 on DCs as a new potential target for improving response rates in patients undergoing immune checkpoint blockade therapy, and CD5 offers great potential for the development of related new drugs (21). All of the above points to its potential prognostic value (21, 60, 61). However, this has not been reported in any previous study of TPVR. EN-RAGE, also known as S100A12, is a calcium-binding pro-inflammatory protein secreted predominantly by activated granulocytes, macrophages, and lymphocytes (62). It has been an important modulator of the inflammatory immune response by acting on RAGE-mediated downstream cascades. Increased levels of EN-RAGE have been reported in the serum, aqueous humor, and tear fluid of autoimmune uveitis patients (63, 64). A previous study found that the RAGE axis was enhanced in PDR and PVR, but the specific mechanisms were unclear (65). In our Olink protein study, we found a robust upregulation and the highest AUC value of EN-RAGE in the TPVR group. In addition, it was positively correlated with IL-8, which was an important cytokine in TPVR (9, 13). Taken together, EN-RAGE may be a potential biomarker for the diagnosis and the prognosis of TPVR.

In addition to identifying effective biomarkers, determining the optimal timing of intervention is another important aspect of improving the prognosis of TPVR. However, the continuous expression changes of biomarkers during the formation of TPVR have been difficult to obtain due to the limited schedule of vitrectomy in clinical practice. The PPI network suggests that interleukin family members may be hub proteins in TPVR formation. Based on the above, IL-6, IL-7, and IL-33 were selected for further validation by ELISA in the rabbit model at different time points during 28 days after injury, and the trend was consistent with the proteomics results from Olink. Notably, IL-6 and IL-33 increased and peaked 1 day after injury and remained high until 28 days, demonstrating early, extensive and persistent post-injury inflammation in the TPVR process. IL-7 expression levels also exhibited a dramatic change 1 day after injury, suggesting that early post-injury may be an optimal time for intervention targeting interleukin family members.

The study provides insights for future clinical management of TPVR. The identification of multiple biomarkers that cover parameters associated with PVR severity and PVR prognosis may create additional avenues for improving diagnostic procedures and disease monitoring, as well as providing clues to appropriate targets for further therapeutic drug discovery. The combined testing of biomarkers that play different roles during this process may prove useful for clinical decision-making, especially for those who cannot achieve vitrectomy at the optimal time, an additional intervention may take action. On the other hand, the robust differential expression of interleukin in the TPVR rabbit model at an early stage of injury validates and demonstrates an excessive inflammatory response soon after injury. This reminds us that early intraocular or systemic prophylactic application of anti-inflammatory therapy can be applied to control the intraocular inflammation at an early stage, thereby inhibiting the development and severity of PVR and ultimately restoring as much retinal function as possible.

## 5 Conclusion

In conclusion, we observed the variation of a wide range of cytokines in the vitreous of TPVR, among which IL-7, EN-RAGE, TWEAK, CD-5, CXCL-5, and IL-33 were our novel findings by comprehensive bioinformatics analysis, providing a preliminary reference for studying the mechanism of TPVR formation. In the future, we will expand clinical sample sizes, improve the diagnostic accuracy of candidate biomarkers, and validate their potential for clinical application. In addition, the molecular mechanisms of these potential biomarkers in TPVR require further in-depth investigation. Targeted therapy based on the right biomarkers and pathway at the right time can ensure ideal outcomes.

## Data availability statement

The original contributions presented in the study are included in the article/**Supplementary Material**, further inquiries can be directed to the corresponding author/s.

## Ethics statement

The studies involving humans were approved by Regional Ethics Committee of the Tianjin Medical University General Hospital (IRB2023-WZ-204). The studies were conducted in accordance with the local legislation and institutional requirements. The participants provided their written informed consent to participate in this study. The animal studies were approved by Ethics Committee of the Tianjin Medical University General Hospital (IRB2023-DW-126). The studies were conducted in accordance with the local legislation and institutional requirements. Written informed consent was obtained from the owners for the participation of their animals in this study.

## Author contributions

HG: Data curation, Investigation, Methodology, Formal Analysis, Writing – original draft. TW: Data curation, Methodology, Conceptualization, Funding acquisition, Validation, Writing – review & editing. JY: Conceptualization, Data curation, Methodology, Writing – review & editing, Supervision. ZS: Conceptualization, Data curation, Writing – review & editing, Formal Analysis, Visualization. ML: Data curation, Writing – review & editing, Methodology. SC: Data curation, Methodology, Writing – review & editing. TH: Methodology, Writing – review & editing, Conceptualization, Investigation, Supervision. HY: Conceptualization, Investigation, Methodology, Supervision, Writing – review & editing, Data curation, Validation, Visualization.

## References

[1] Dai Y Dai C Sun T. Inflammatory mediators of proliferative vitreoretinopathy: hypothesis and review. Int Ophthalmol. (2020) 40:1587–601. doi: 10.1007/s10792-020-01325-4 PMC7242233 32103371

[2] Pastor JC Rojas J Pastor-Idoate S Di Lauro S Gonzalez-Buendia L Delgado-Tirado S. Proliferative vitreoretinopathy: A new concept of disease pathogenesis and practical consequences. Prog Retin Eye Res. (2016) 51:125–55. doi: 10.1016/j.preteyeres.2015.07.005 26209346

[3] Ferro Desideri L Artemiev D Zandi S Zinkernagel MS Anguita R. Proliferative vitreoretinopathy: an update on the current and emerging treatment options. Graefes Arch Clin Exp Ophthalmol. (2023) 16. doi: 10.1007/s00417-023-06264-1 PMC10907475 37843566

[4] Ricker LJ Kessels AG de Jager W Hendrikse F Kijlstra A la Heij EC. Prediction of proliferative vitreoretinopathy after retinal detachment surgery: potential of biomarker profiling. Am J Ophthalmol. (2012) 154:347–354.e2. doi: 10.1016/j.ajo.2012.02.004 22541653

[5] Morescalchi F Duse S Gambicorti E Romano MR Costagliola C Semeraro F. Proliferative vitreoretinopathy after eye injuries: an overexpression of growth factors and cytokines leading to a retinal keloid. Mediators Inflammation. (2013) 2013:269787. doi: 10.1155/2013/269787 PMC3806231 24198445

[6] Yu J Peng R Chen H Cui C Ba J. Elucidation of the pathogenic mechanism of rhegmatogenous retinal detachment with proliferative vitreoretinopathy by proteomic analysis. Invest Ophthalmol Vis Sci. (2012) 53:8146–53. doi: 10.1167/iovs.12-10079 23139279

[7] Kon CH Occleston NL Aylward GW Khaw PT. Expression of vitreous cytokines in proliferative vitreoretinopathy: a prospective study. Invest Ophthalmol Vis Sci. (1999) 40:705–12. 10067974

[8] Canataroglu H Varinli I Ozcan AA Canataroglu A Doran F Varinli S. Interleukin (IL)-6, interleukin (IL)-8 levels and cellular composition of the vitreous humor in proliferative diabetic retinopathy, proliferative vitreoretinopathy, and traumatic proliferative vitreoretinopathy. Ocul Immunol Inflammation. (2005) 13:375–81. doi: 10.1080/09273940490518900 16422002

[9] Zhou Q Xu G Zhang X Cao C Zhou Z. Proteomics of post-traumatic proliferative vitreoretinopathy in rabbit retina reveals alterations to a variety of functional proteins. Curr Eye Res. (2012) 37:318–26. doi: 10.3109/02713683.2011.635397 22295879

[10] Wang M Li Q Dong H. Proteomic evidence that ABCA4 is vital for traumatic proliferative vitreoretinopathy formation and development. Exp Eye Res. (2019) 181:232–9. doi: 10.1016/j.exer.2019.02.006 30738069

[11] Zhao YM Sun RS Duan F Wang FY Li YJ Qian XB. Intravitreal slow-release dexamethasone alleviates traumatic proliferative vitreoretinopathy by inhibiting persistent inflammation and Müller cell gliosis in rabbits. Int J Ophthalmol. (2023) 16:22–32. doi: 10.18240/ijo.2023.01.04 36659954 PMC9815969

[12] Nashed A Saikia P Herrmann WA Gabel VP Helbig H Hillenkamp J. The outcome of early surgical repair with vitrectomy and silicone oil in open-globe injuries with retinal detachment. Am J Ophthalmol. (2011) 151:522–8. doi: 10.1016/j.ajo.2010.08.041 21168826

[13] Haslam DE Li J Dillon ST Gu X Cao Y Zeleznik OA. Stability and reproducibility of proteomic profiles in epidemiological studies: comparing the Olink and SOMAscan platforms. Proteomics. (2022) 22:e2100170. doi: 10.1002/pmic.202100170 35598103 PMC9923770

[14] Wang X Yip KC He A Tang J Liu S Yan R. Plasma olink proteomics identifies CCL20 as a novel predictive and diagnostic inflammatory marker for preeclampsia. J Proteome Res. (2022) 21:2998–3006. doi: 10.1021/acs.jproteome.2c00544 36301636 PMC9724708

[15] Guo H Yu J He T Chen S Sun Z Zhang J. Early use of intravitreal triamcinolone to inhibit traumatic proliferative vitreoretinopathy: a randomised clinical trial. Br J Ophthalmol. (2023), bjo–2023-324318. doi: 10.1136/bjo-2023-324318 38041678

[16] Long Y Li Y Wang T Ni A Guo J Dong Q. Inflammation-related proteomics demonstrate landscape of fracture blister fluid in patients with acute compartment syndrome. Front Immunol. (2023) 14:1161479. doi: 10.3389/fimmu.2023.1161479 37090725 PMC10115951

[17] Chen XF Du M Wang XH Yan H. Effect of etanercept on post-traumatic proliferative vitreoretinopathy. Int J Ophthalmol. (2019) 12:731–8. doi: 10.18240/ijo.2019.05.06 PMC6520276 31131230

[18] Kuo PL Huang MS Hung JY Chou SH Chiang SY Huang YF. Synergistic effect of lung tumor-associated dendritic cell-derived HB-EGF and CXCL5 on cancer progression. Int J Cancer. (2014) 135:96–108. doi: 10.1002/ijc.28673 24346967

[19] Wang C Kong L Kim S Lee S Oh S Jo S. The role of IL-7 and IL-7R in cancer pathophysiology and immunotherapy. Int J Mol Sci. (2022) 23:10412. doi: 10.3390/ijms231810412 36142322 PMC9499417

[20] Dwyer BJ Jarman EJ Gogoi-Tiwari J Ferreira-Gonzalez S Boulter L Guest RV. TWEAK/Fn14 signalling promotes cholangiocarcinoma niche formation and progression. J Hepatol. (2021) 74:860–72. doi: 10.1016/j.jhep.2020.11.018 33221352

[21] Voisinne G Gonzalez de Peredo A Roncagalli R. CD5, an undercover regulator of TCR signaling. Front Immunol. (2018) 9:2900. doi: 10.3389/fimmu.2018.02900 30581443 PMC6292949

[22] Korets SB Czok S Blank SV Curtin JP Schneider RJ. Targeting the mTOR/4E-BP pathway in endometrial cancer. Clin Cancer Res. (2011) 17:7518–28. doi: 10.1158/1078-0432.CCR-11-1664 22142830

[23] Symeonidis C Papakonstantinou E Androudi S Georgalas I Rotsos T Karakiulakis G. Comparison of interleukin-6 and matrix metalloproteinase expression in the subretinal fluid and the vitreous during proliferative vitreoretinopathy: correlations with extent, duration of RRD and PVR grade. Cytokine. (2014) 67:71–6. doi: 10.1016/j.cyto.2014.02.012 24725542

[24] El-Ghrably IA Dua HS Orr GM Fischer D Tighe PJ. Intravitreal invading cells contribute to vitreal cytokine milieu in proliferative vitreoretinopathy. Br J Ophthalmol. (2001) 85:461–70. doi: 10.1136/bjo.85.4.461 PMC1723908 11264138

[25] Roybal CN Velez G Toral MA Tsang SH Bassuk AG Mahajan VB. Personalized proteomics in proliferative vitreoretinopathy implicate hematopoietic cell recruitment and mTOR as a therapeutic target. Am J Ophthalmol. (2018) 186:152–63. doi: 10.1016/j.ajo.2017.11.025 PMC5805631 29246578

[26] Kauffmann DJ van Meurs JC Mertens DA Peperkamp E Master C Gerritsen ME. Cytokines in vitreous humor: interleukin-6 is elevated in proliferative vitreoretinopathy. Invest Ophthalmol Vis Sci. (1994) 35:900–6. 8125753

[27] Chen X Yang W Deng X Ye S Xiao W. Interleukin-6 promotes proliferative vitreoretinopathy by inducing epithelial-mesenchymal transition *via* the JAK1/STAT3 signaling pathway. Mol Vis. (2020) 26:517–29. PMC7406861 32818015

[28] Zhao Y Wei K Chi H Xia Z Li X. IL-7: A promising adjuvant ensuring effective T cell responses and memory in combination with cancer vaccines? Front Immunol. (2022) 13:1022808. doi: 10.3389/fimmu.2022.1022808 36389666 PMC9650235

[29] ElKassar N Gress RE. An overview of IL-7 biology and its use in immunotherapy. J Immunotoxicol. (2010) 7:1–7. doi: 10.3109/15476910903453296 20017587 PMC2826542

[30] Lundström W Fewkes NM Mackall CL. IL-7 in human health and disease. Semin Immunol. (2012) 24:218–24. doi: 10.1016/j.smim.2012.02.005 PMC3358500 22410365

[31] Gao R Zhou P Li Y Li Q. High glucose-induced IL-7/IL-7R upregulation of dermal fibroblasts inhibits angiogenesis in a paracrine way in delayed diabetic wound healing. J Cell Commun Signal. (2023) 17:1023–38. doi: 10.1007/s12079-023-00754-x PMC10409704 37217704

[32] Bartlett A Sanders AJ Ruge F Harding KG Jiang WG. Potential implications of interleukin-7 in chronic wound healing. Exp Ther Med. (2016) 12:33–40. doi: 10.3892/etm.2016.3263 27347014 PMC4906893

[33] Huang M Sharma S Zhu LX Keane MP Luo J Zhang L. IL-7 inhibits fibroblast TGF-beta production and signaling in pulmonary fibrosis. J Clin Invest. (2002) 109:931–7. doi: 10.1172/JCI14685 PMC150933 11927620

[34] Zhang L Keane MP Zhu LX Sharma S Rozengurt E Strieter RM. Interleukin-7 and transforming growth factor-beta play counter-regulatory roles in protein kinase C-delta-dependent control of fibroblast collagen synthesis in pulmonary fibrosis. J Biol Chem. (2004) 279:28315–9. doi: 10.1074/jbc.C400115200 15133032

[35] Kim SY Yeo A Noh H Ji YW Song JS Kim HC. Downregulation of IL-7 and IL-7R reduces membrane-type matrix metalloproteinase 14 in granular corneal dystrophy type 2 keratocyte. Invest Ophthalmol Vis Sci. (2018) 59:5693–703. doi: 10.1167/iovs.18-25161 30489629

[36] Cayrol C Girard JP. Interleukin-33 (IL-33): A nuclear cytokine from the IL-1 family. Immunol Rev. (2018) 281:154–68. doi: 10.1111/imr.12619 29247993

[37] Xi H Katschke KJ Jr Li Y Truong T Lee WP Diehl L. IL-33 amplifies an innate immune response in the degenerating retina. J Exp Med. (2016) 213:189–207. doi: 10.1084/jem.20150894 26755704 PMC4749925

[38] Liu XC Liu XF Jian CX Li CJ He SZ. IL-33 is induced by amyloid-β stimulation and regulates inflammatory cytokine production in retinal pigment epithelium cells. Inflammation. (2012) 35:776–84. doi: 10.1007/s10753-011-9379-4 21898270

[39] Sugita J Asada Y Kawano H. The role of interleukin-33 expression in retinal tissue. Investig Ophthalmol Vis Sci. (2014) 55:708.

[40] Halát G Haider T Dedeyan M Heinz T Hajdu S Negrin LL. IL-33 and its increased serum levels as an alarmin for imminent pulmonary complications in polytraumatized patients. World J Emerg Surg. (2019) 14:36. doi: 10.1186/s13017-019-0256-z 31360218 PMC6642565

[41] Theodoropoulou S Copland DA Liu J Dick AD. Role of interleukin 33/ST2 axis in the immune-mediated pathogenesis of age-related macular degeneration. Lancet. (2015) 385 Suppl 1:S97. doi: 10.1016/S0140-6736(15)60412-3 26312920

[42] Pichery M Mirey E Mercier P Lefrancais E Dujardin A Ortega N. Endogenous IL-33 is highly expressed in mouse epithelial barrier tissues, lymphoid organs, brain, embryos, and inflamed tissues: in *situ* analysis using a novel Il-33-LacZ gene trap reporter strain. J Immunol. (2012) 188:3488–95. doi: 10.4049/jimmunol.1101977 22371395

[43] Theodoropoulou S Copland DA Liu J Wu J Gardner PJ Ozaki E. Interleukin-33 regulates tissue remodelling and inhibits angiogenesis in the eye. J Pathol. (2017) 241:45–56. doi: 10.1002/path.4816 27701734 PMC5683707

[44] Adachi K Hirakata T Asada Y Iwamoto S Nakae S Matsuda A. The role of interleukin-33 in retinal tissue fibrosis after laser injury. Investig Ophthalmol Vis Sci. (2018) 59:344.

[45] Aksünger A Or M Okur H Hasanreisoğlu B Akbatur H. Role of interleukin 8 in the pathogenesis of proliferative vitreoretinopathy. Ophthalmologica. (1997) 211:223–5. doi: 10.1159/000310794 9216011

[46] Yoshida A Elner SG Bian ZM Elner VM. Induction of interleukin-8 in human retinal pigment epithelial cells after denuding injury. Br J Ophthalmol. (2001) 85:872–6. doi: 10.1136/bjo.85.7.872 PMC1724031 11423465

[47] Walz A Burgener R Car B Baggiolini M Kunkel SL Strieter RM. Structure and neutrophil-activating properties of a novel inflammatory peptide (ENA-78) with homology to interleukin 8. J Exp Med. (1991) 174:1355–62. doi: 10.1084/jem.174.6.1355 PMC2119025 1744577

[48] Persson T Monsef N Andersson P Bjartell A Malm J Calafat J. Expression of the neutrophil-activating CXC chemokine ENA-78/CXCL5 by human eosinophils. Clin Exp Allergy. (2003) 33:531–7. doi: 10.1046/j.1365-2222.2003.01609.x 12680872

[49] Mei J Liu Y Dai N Hoffmann C Hudock KM Zhang P. Cxcr2 and Cxcl5 regulate the IL-17/G-CSF axis and neutrophil homeostasis in mice. J Clin Invest. (2012) 122:974–86. doi: 10.1172/JCI60588 PMC3287232 22326959

[50] Kuo PL Chen YH Chen TC Shen KH Hsu YL. CXCL5/ENA78 increased cell migration and epithelial-to-mesenchymal transition of hormone-independent prostate cancer by early growth response-1/snail signaling pathway. J Cell Physiol. (2011) 226:1224–31. doi: 10.1002/jcp.22445 20945384

[51] Zandi S Pfister IB Garweg JG. Postoperative proliferative vitreoretinopathy development is linked to vitreal CXCL5 concentrations. Sci Rep. (2021) 11:23989. doi: 10.1038/s41598-021-03294-9 34907233 PMC8671512

[52] Poveda J Vázquez-Sánchez S Sanz AB Ortiz A Ruilope LM Ruiz-Hurtado G. TWEAK-Fn14 as a common pathway in the heart and the kidneys in cardiorenal syndrome. J Pathol. (2021) 254:5–19. doi: 10.1002/path.5631 33512736

[53] Ratajczak W Atkinson SD Kelly C. The TWEAK/Fn14/CD163 axis-implications for metabolic disease. Rev Endocr Metab Disord. (2022) 23:449–62. doi: 10.1007/s11154-021-09688-4 PMC9156485 34542797

[54] Short C Zhong A Xu J Mahdi E Glazier A Malkoff N. TWEAK/FN14 promotes profibrogenic pathway activation in Prominin-1-expressing hepatic progenitor cells in biliary atresia. Hepatology. (2023) 77:1639–53. doi: 10.1097/HEP.0000000000000026 36626628

[55] Pascoe AL Johnston AJ Murphy RM. Controversies in TWEAK-Fn14 signaling in skeletal muscle atrophy and regeneration. Cell Mol Life Sci. (2020) 77:3369–81. doi: 10.1007/s00018-020-03495-x PMC11104974 32200423

[56] Liu J Liu Y Peng L Li J Wu K Xia L. TWEAK/fn14 signals mediate burn wound repair. J Invest Dermatol. (2019) 139:224–34. doi: 10.1016/j.jid.2018.05.036 30081003

[57] Ameri H Liu H Liu R Ha Y Paulucci-Holthauzen AA Hu S. TWEAK/Fn14 pathway is a novel mediator of retinal neovascularization. Invest Ophthalmol Vis Sci. (2014) 55:801–13. doi: 10.1167/iovs.13-12812 PMC3920863 24408972

[58] Cheadle L Rivera SA Phelps JS Ennis KA Stevens B Burkly LC. Sensory experience engages microglia to shape neural connectivity through a non-phagocytic mechanism. Neuron. (2020) 108:451–468.e9. doi: 10.1016/j.neuron.2020.08.002 32931754 PMC7666095

[59] Chen DY Su GF. Tumor necrosis factor-like weak inducer of apoptosis association with proliferative diabetic retinopathy and promotes proliferation and collagen synthesis in retinal ARPE-19 cells. Genet Mol Res. (2016) 15. doi: 10.4238/gmr.15016920 27050952

[60] Schwarz S Linnebacher M. CD5: from antiquated T cell marker to immunotherapy's new hope. Signal Transduct Target Ther. (2023) 8:216. doi: 10.1038/s41392-023-01494-5 37230972 PMC10212910

[61] He M Roussak K Ma F Borcherding N Garin V White M. CD5 expression by dendritic cells directs T cell immunity and sustains immunotherapy responses. Science. (2023) 379:eabg2752. doi: 10.1126/science.abg2752 36795805 PMC10424698

[62] Meijer B Gearry RB Day AS. The role of S100A12 as a systemic marker of inflammation. Int J Inflam. (2012) 2012:907078. doi: 10.1155/2012/907078 22811950 PMC3395136

[63] Angeles-Han ST Utz VM Thornton S Schulert G Rodriguez-Smith J Kauffman A. S100 proteins, cytokines, and chemokines as tear biomarkers in children with juvenile idiopathic arthritis-associated uveitis. Ocul Immunol Inflammation. (2021) 29:1616–20. doi: 10.1080/09273948.2020.1758731 PMC8842740 35169380

[64] Mahaling B Low SWY Beck M Kumar D Ahmed S Connor TB. Damage-associated molecular patterns (DAMPs) in retinal disorders. Int J Mol Sci. (2022) 23:2591. doi: 10.3390/ijms23052591 35269741 PMC8910759

[65] Pachydaki SI Tari SR Lee SE Ma W Tseng JJ Sosunov AA. Upregulation of RAGE and its ligands in proliferative retinal disease. Exp Eye Res. (2006) 82:807–15. doi: 10.1016/j.exer.2005.09.022 16364297

