# Supplementary material for: Vitreous Olink proteomics reveals inflammatory biomarkers for diagnosis and prognosis of traumatic proliferative vitreoretinopathy
Source: Front Immunol. 2024 Feb 22;15:1355314. doi: 10.3389/fimmu.2024.1355314 (PMC10917961; doi:10.3389/fimmu.2024.1355314)
Supplement: Supplementary file 1 [file DataSheet_1.pdf]

olink® | INFLAMMATION

ARTICLE NUMBER: 95302

Adenosine Deaminase (ADA)	P00813	Fractalkine (CX3CL1)	P78423
Artemin (ARTN)	Q5T4W7	Glial cell line-derived neurotrophic factor (GDNF)	P39905
Axin-1 (AXIN1)	O15169	Hepatocyte growth factor (HGF)	P14210
Beta-nerve growth factor (Beta-NGF)	P01138	Interferon gamma (IFN-gamma)	P01579
Caspase-8 (CASP-8)	Q14790	Interleukin-1 alpha (IL-1 alpha)	P01583
C-C motif chemokine 3 (CCL3)	P10147	Interleukin-2 (IL-2)	P60568
C-C motif chemokine 4 (CCL4)	P13236	Interleukin-2 receptor subunit beta (IL-2RB)	P14784
C-C motif chemokine 19 (CCL19)	Q99731	Interleukin-4 (IL-4)	P05112
C-C motif chemokine 20 (CCL20)	P78556	Interleukin-5 (IL5)	P05113
C-C motif chemokine 23 (CCL23)	P55773	Interleukin-6 (IL6)	P05231
C-C motif chemokine 25 (CCL25)	O15444	Interleukin-7 (IL-7)	P13232
C-C motif chemokine 28 (CCL28)	Q9NRJ3	Interleukin-8 (IL-8)	P10145
CD40L receptor (CD40)	P25942	Interleukin-10 (IL10)	P22301
CUB domain-containing protein 1 (CDCP1)	Q9H5V8	Interleukin-10 receptor subunit alpha (IL-10RA)	Q13651
C-X-C motif chemokine 1 (CXCL1)	P09341	Interleukin-10 receptor subunit beta (IL-10RB)	Q08334
C-X-C motif chemokine 5 (CXCL5)	P42830	Interleukin-12 subunit beta (IL-12B)	P29460
C-X-C motif chemokine 6 (CXCL6)	P80162	Interleukin-13 (IL-13)	P35225
C-X-C motif chemokine 9 (CXCL9)	Q07325	Interleukin-15 receptor subunit alpha (IL-15RA)	Q13261
C-X-C motif chemokine 10 (CXCL10)	P02778	Interleukin-17A (IL-17A)	Q16552
C-X-C motif chemokine 11 (CXCL11)	O14625	Interleukin-17C (IL-17C)	Q9P0M4
Cystatin D (CST5)	P28325	Interleukin-18 (IL-18)	Q14116
Delta and Notch-like epidermal growth factor-related receptor (DNER)	Q8NFT8	Interleukin-18 receptor 1 (IL-18R1)	Q13478
Eotaxin (CCL11)	P51671	Interleukin-20 (IL-20)	Q9NYY1
Eukaryotic translation initiation factor 4E-binding protein 1 (4E-BP1)	Q13541	Interleukin-20 receptor subunit alpha (IL-20RA)	Q9UHF4
Fibroblast growth factor 21 (FGF-21)	Q9NSA1	Interleukin-22 receptor subunit alpha-1 (IL-22 RA1)	Q8N6P7
Fibroblast growth factor 23 (FGF-23)	Q9GZV9	Interleukin-24 (IL-24)	Q13007
Fibroblast growth factor 5 (FGF-5)	P12034	Interleukin-33 (IL-33)	O95760
Fibroblast growth factor 19 (FGF-19)	O95750	Latency-associated peptide transforming growth factor beta-1 (LAP TGF-beta-1)	P01137
Fms-related tyrosine kinase 3 ligand (Flt3L)	P49771	Leukemia inhibitory factor (LIF)	P15018

Table continues on reverse ▶

Leukemia inhibitory factor receptor (LIF-R)	P42702	STAM-binding protein (STAMBP)	O95630
Macrophage colony-stimulating factor 1 (CSF-1)	P09603	Stem cell factor (SCF)	P21583
Matrix metalloproteinase-1 (MMP-1)	P03956	Sulfotransferase 1A1 (ST1A1)	P50225
Matrix metalloproteinase-10 (MMP-10)	P09238	T cell surface glycoprotein CD6 isoform (CD6)	P30203
Monocyte chemotactic protein 1 (MCP-1)	P13500	T-cell surface glycoprotein CD5 (CD5)	P06127
Monocyte chemotactic protein 2 (MCP-2)	P80075	T-cell surface glycoprotein CD8 alpha chain (CD8A)	P01732
Monocyte chemotactic protein 3 (MCP-3)	P80098	Thymic stromal lymphopoietin (TSLP)	Q969D9
Monocyte chemotactic protein 4 (MCP-4)	Q99616	TNF-beta (TNFB)	P01374
Natural killer cell receptor 2B4 (CD244)	Q9BZW8	TNF-related activation-induced cytokine (TRANCE)	O14788
Neurotrophin-3 (NT-3)	P20783	TNF-related apoptosis-inducing ligand (TRAIL)	P50591
Neurturin (NRTN)	Q99748	Transforming growth factor alpha (TGF-alpha)	P01135
Oncostatin-M (OSM)	P13725	Tumor necrosis factor (Ligand) superfamily, member 12 (TWEAK)	O43508
Osteoprotegerin (OPG)	O00300	Tumor necrosis factor (TNF)	P01375
Programmed cell death 1 ligand 1 (PD-L1)	Q9NZQ7	Tumor necrosis factor ligand superfamily member 14 (TNFSF14)	O43557
Protein S100-A12 (EN-RAGE)	P80511	Tumor necrosis factor receptor superfamily member 9 (TNFRSF9)	Q07011
Signaling lymphocytic activation molecule (SLAMF1)	Q13291	Urokinase-type plasminogen activator (uPA)	P00749
SIR2-like protein 2 (SIRT2)	Q8IXJ6	Vascular endothelial growth factor A (VEGF-A)	P15692

Biological Process

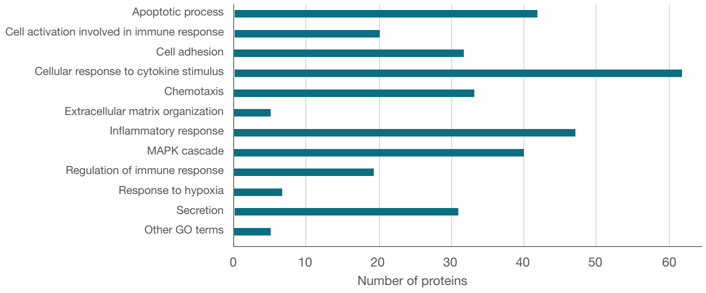

Disease Area

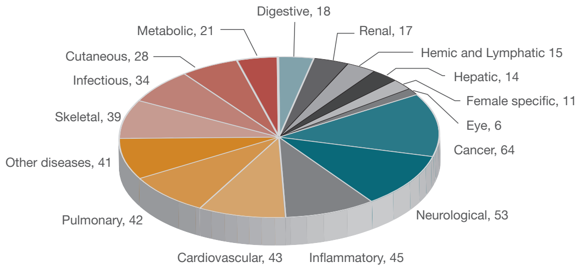

Classification of biomarker proteins included in the panel based on Biological Process and Disease Area. Note that proteins may belong to multiple classes in some cases.

For more details visit **www.olink.com/inflammation**

1029 v1.3, 2019-03-29

This product is sold under license from PHRI Properties, Inc. and may be used under the PHRI Properties patent rights outside the field of human *in vitro* diagnostics. Olink is a registered trademark of Olink Proteomics AB. All third party trademarks are the property of their respective owners.

© 2016–2019 Olink Proteomics AB.

Olink Proteomics AB, Uppsala Science Park, SE-751 83 Uppsala, Sweden
Olink Proteomics Inc., 201 Dexter Ave, Watertown, MA 02472, USA

www.olink.com
