# Supplementary material for: Vitreous Olink proteomics reveals inflammatory biomarkers for diagnosis and prognosis of traumatic proliferative vitreoretinopathy
Source: Front Immunol. 2024 Feb 22;15:1355314. doi: 10.3389/fimmu.2024.1355314 (PMC10917961; doi:10.3389/fimmu.2024.1355314)
Supplement: Supplementary file 2 [file Table_1.docx]

TABLE 1. TPVR grading and scoring criteria

| Grading | Retinopathy | Scoring |
| --- | --- | --- |
| 0 | Normal retinal | 14 |
| I | Retina detached, but surface was smooth | 13 |
| II | Retinal detachment with unsmooth epiretinal or subretinal, but no proliferative membrane |  |
| II-a | Present in 1 quadrant | 12 |
| II-b | Present in 2 quadrants | 11 |
| II-c | Present in 3 quadrants | 10 |
| II-d | Present in 4 quadrants | 9 |
| III | Retinal detachment with epiretinal or subretinal proliferative membrane |  |
| III-a | Present in 1 quadrant | 8 |
| III-b | Present in 2 quadrants | 7 |
| III-c | Present in 3 quadrants | 6 |
| III-d | Present in 4 quadrants | 5 |
| IV | Retinal detachment with wrinkling of the retina |  |
| IV-a | Present in 1 quadrant | 4 |
| IV-b | Present in 2 quadrants | 3 |
| IV-c | Present in 3 quadrants | 2 |
| IV-d | Present in 4 quadrants | 1 |
| TPVR= traumatic proliferative vitreoretinopathy | | |
