# Supplementary material for: Vitreous Olink proteomics reveals inflammatory biomarkers for diagnosis and prognosis of traumatic proliferative vitreoretinopathy
Source: Front Immunol. 2024 Feb 22;15:1355314. doi: 10.3389/fimmu.2024.1355314 (PMC10917961; doi:10.3389/fimmu.2024.1355314)
Supplement: Supplementary file 8 [file Table_7.docx]

Table 4. The expression level of IL-6 and IL-33 at 0, 1, 3, 7, 10, 14, 28 days after injury

| N=5 | IL-33 (pg/ml) | IL-6 (pg/ml) | IL-7 (pg/ml) |
| --- | --- | --- | --- |
| Control | 72.15-93.77 (85.07±8.11) | 51.83-58.49 (55.38±2.98) | 32.38-39.48 (36.03 ± 2.77) |
| Baseline | 102.96-166.77 (125.54±23.71) | 45.12-68.04 (57.82±8) | 21.96-38.32 (32.84 ± 5.64) |
| 1 day post-injury | **222.73-269.46 (239.05±18.28)** | **91.2-107.15 (95.3±6.72)** | **17.24-22.46 (18.71 ± 1.93)** |
| 3 days post-injury | 172.22-231.22 (202.04±25.73) | 71.42-101.24 (85.73±10.89) | 33.09-43.76 (36.61 ± 3.72) |
| 7 days post-injury | 168.46-226.19 (202.98±21.35) | 68.35-99.84 (83.54±11.48) | 25.62-40.81 (30.26 ± 5.55) |
| 10 days post-injury | 176.87-208.5 (189.02±13.52) | 75.98-95.23 (85.7±6.82) | 19.24-25.94 (22.49 ± 2.74) |
| 14 days post-injury | 191.39-206.94 (199.53 ± 5.27) | 70.57-94.37 (79.78±10.81) | 25.22-33.90 (29.51 ± 3.51) |
| 28 days post-injury | 178.02-231.94 (202.9±24.21) | 66.94-84.36 (75.97±7.67) | 38.22-43.83 (40.66 ± 2.32) |
